# Somatic drivers of B-ALL in a model of *ETV6-RUNX1; Pax5*^*+/−*^ leukemia

**DOI:** 10.1186/s12885-015-1586-1

**Published:** 2015-08-13

**Authors:** Louise van der Weyden, George Giotopoulos, Kim Wong, Alistair G. Rust, Carla Daniela Robles-Espinoza, Hikari Osaki, Brian J. Huntly, David J. Adams

**Affiliations:** 1 Wellcome Trust Sanger Institute, Wellcome Trust Genome Campus, Hinxton, Cambridge, CB10 1HH UK; 2 Cambridge Institute for Medical Research and Wellcome Trust/MRC Cambridge Stem Cell Institute, Wellcome Trust/MRC Building, Addenbrooke’s Hospital, Hills Road, Cambridge, CB2 0XY UK; 3 Experimental Cancer Genetics, Wellcome Trust Sanger Institute, Wellcome Trust Genome Campus, Hinxton, Cambridge, CB10 1HH UK

**Keywords:** *ETV6-RUNX1*, *Pax5*, *JAK/STAT*, *Trp53*, Leukemia, B-cell precursor, Insertional mutagenesis

## Abstract

**Background:**

B-cell precursor acute lymphoblastic leukemia (B-ALL) is amongst the leading causes of childhood cancer-related mortality. Its most common chromosomal aberration is the *ETV6-RUNX1* fusion gene, with ~25 % of *ETV6-RUNX1* patients also carrying *PAX5* alterations.

**Methods:**

We have recreated this mutation background by inter-crossing *Etv6-RUNX1* (*Etv6*^*RUNX1-SB*^) and *Pax5*^*+/−*^ mice and performed an *in vivo* analysis to find driver genes using *Sleeping Beauty* transposon-mediated mutagenesis and also exome sequencing.

**Results:**

Combination of *Etv6-RUNX1* and *Pax5*^*+/−*^ alleles generated a transplantable B220 + CD19+ B-ALL with a significant disease incidence. RNA-seq analysis showed a gene expression pattern consistent with arrest at the pre-B stage. Analysis of the transposon common insertion sites identified genes involved in B-cell development (*Zfp423*) and the JAK/STAT signaling pathway (*Jak1, Stat5* and *Il2rb*), while exome sequencing revealed somatic hotspot mutations in *Jak1* and *Jak3* at residues analogous to those mutated in human leukemias, and also mutation of *Trp53*.

**Conclusions:**

Powerful synergies exists in our model suggesting STAT pathway activation and mutation of *Trp53* are potent drivers of B-ALL in the context of *Etv6-RUNX1;Pax5*^*+/−*^*.*

**Electronic supplementary material:**

The online version of this article (doi:10.1186/s12885-015-1586-1) contains supplementary material, which is available to authorized users.

## Background

B-cell precursor acute lymphoblastic leukemia (B-ALL) is the most common childhood tumor [1]. The most common chromosomal rearrangement in B-ALL is the t(12;21)(p13;q22) translocation generating the *ETV6*-*RUNX1* fusion gene [2]. This fusion is necessary but insufficient for the development of B-ALL, as monozygotic twin studies, and the detection of the *ETV6*-*RUNX1* fusion in fetal blood spots from patients who do not go on to develop B-ALL have shown [3, 4].

*PAX5,* a guardian of B-cell identity and function, is somatically mutated in ~40 % of cases of childhood B-ALL [5]. Moreover, the most common recurrent focal deletion region in *ETV6*-*RUNX1+* tumors involves *PAX5* (9p13.2; 25 %) and these deletions are thought to be early events in leukemogenesis [6]. Previously, we generated a knock-in mouse model of *ETV6-RUNX1* ALL, in which expression of the fusion gene is driven from the endogenous *Etv6* promoter, and is linked to expression of the *Sleeping Beauty* (SB) transposase allowing the identification of transposon gene mutations that co-operate with *Etv6-RUNX1* in leukemia development [7]. Given that *PAX5* heterozygosity is a frequent event in *ETV6-RUNX1* patients [5], we bred these mice onto a background of *Pax5* heterozygosity and performed a *SB* transposon-mediated mutagenesis screen to explore the profile of co-operating drivers. We coupled this approach with targeted exome sequencing of tumors to find additional mutations, and in particular hotspot mutation events.

## Methods

### Mouse strains

Generation and genotyping of *Etv6-RUNX1, T2Onc* [7] and *Pax5* [8] mice has been described previously. For secondary bone marrow transplants of tumors, 6–12 week old SCID mice were inoculated with 3.5-5 × 10^5^ bone marrow or spleen cells by tail vein injection. Animal studies were approved by the Home Office UK. Flow cytometric analysis of CD antigen expression was performed on single-cell suspensions from spleen or bone marrow as described previously [7].

### Identification and analysis of genes affected by SB mutagenesis

Isolation of the transposon insertion sites and Gaussian kernel convolution statistical methods to identify common insertion sites (CISs) have been described previously [7]. Whole transcriptome sequencing (RNA-seq) was performed on splenic RNA using the mRNA Seq Sample Prep Kit (Illumina, San Diego, CA) to create libraries that were sequenced on the Illumina platform. HTSeq-counts (HTSeq framework; v0.54p5) were used as input to edgeR (v3.4.2). Genes with significant differential expression were defined based on an FDR of 5 %. Pathway and gene set enrichment analysis (GSEA) was performed using Ingenuity Pathway Analysis and GSEA (v2.0.14), respectively.

### Exome sequencing and bait design

Spleen (‘tumor’) and tail (‘normal’) genomic DNA were extracted using the Gentra Puregene Cell Kit (Qiagen). Exon-coding sequences of genes previously found to be involved in cancer were captured using custom-designed baits (Additional file 1) and sequenced on an Illumina platform. For each tumor-normal pair, MuTect (v1.14) was used to identify somatic SNVs, which were annotated using the Variant Effect Predictor tool (Ensembl v74). The *Jak1*, *Jak3* and *Trp53* mutations were validated by capillary sequencing.

## Results and discussion

**Fig. 1:**
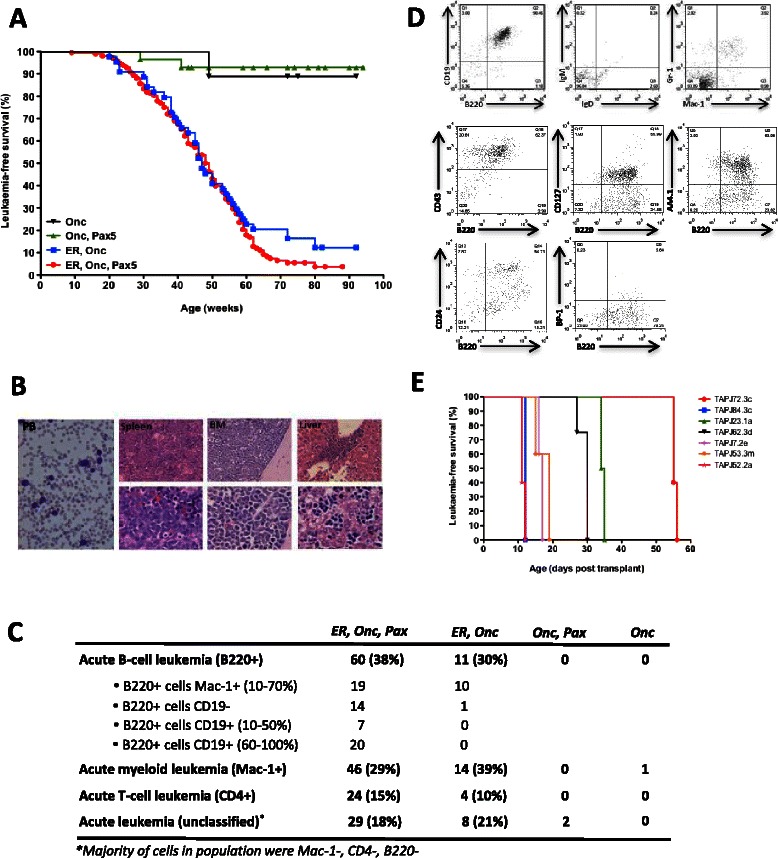
Phenotype of *Etv6-RUNX1, T2Onc, Pax5* leukemias. **a** Kaplan-Meier curves showing the tumor latency of ‘jumping’ *Etv6*^*+/RUNX1*^, *T2Onc*^*+/Tg*^, *Pax5*^*+/−*^ (*ER, Onc, Pax*) and *Etv6*^*+/RUNX1*^, *T2Onc*^*+/Tg*^ (*ER, Onc*) mice, and ‘non-jumping’ control mice (*Onc, Pax* and *Onc*). **b** The presence of lymphoblasts in the peripheral blood (PB), spleen, bone marrow and liver from a representative mouse with leukemia. Magnification: peripheral blood smear (×400), upper row organs (×400) and lower row organs (×1,000). **c** Classification of the leukemias developed by mice shown in the Kaplan-Meier curve according to the Bethesda criteria for lymphoid and non-lymphoid murine malignancies. **d** Upper row: FACS plots from the bone marrow of a representative mouse demonstrate only background Gr-1/Mac-1 myeloid cells, with the majority of cells having a B220+/CD19+/surface Ig-. Middle and lower row: FACS plots from the bone marrow of a representative mouse demonstrate the B220+ cells to have a CD43+, CD127+, AA4.1+, CD24+, BP-1- phenotype. **e** Survival curves for SCID mice transplanted with 3.5-5 × 10^5^ B220+, CD19+ leukemia cells from *ER, Onc, Pax* mice and TAPJ23.1a which was an *ER, Pax5* mouse. Each color represents a different ‘primary’ leukemia, as indicated by the “TAPJ” name of the mouse (*n* = 7)

To perform the *SB* transposon-mediated mutagenesis screen we intercrossed *Pax5* (*Pax5*^*+/−*^) mice with transposon-carrying *T2Onc* mice and the resulting offspring were intercrossed with transposase-carrying *Etv6-RUNX1* (*Etv6*^*+/RUNX1-SB*^) mice (Methods). The resulting genotypes in which transposition would occur were *Etv6*^*+/RUNX1-SB*^, *T2Onc*^*+/Tg*^, *Pax5*^+/−^ (hereafter referred to as *ER, Onc, Pax*) and *Etv6*^*+/RUNX1-SB*^, *T2Onc*^*+/Tg*^ mice (hereafter referred to as *ER, Onc*). Importantly we found that *ER, Onc, Pax* mice showed a significant increase in the proportion of B-cell precursor (BCP)-ALL cases when compared to *ER, Onc* mice wildtype for *Pax5* (41/159 (26 %) versus 1/37 (3 %); p < 0.005 using a 2-tailed Fisher’s exact test), with 27/41 (66 %) of these cases being B220+ CD19+ (Fig. 1). Additional immunophenotyping of these B220+ CD19+ cells from *ER, Onc, Pax* mice confirmed their ontogenic arrest at the pre-B stage (consistent with Hardy fraction C’/D and *ETV6-RUNX1*+ patient leukemic cells; Fig. 1d). Importantly, the leukemias with an almost pure population of B220 + CD19+ cells were also transplantable in SCID mice (with recipients developing B-ALL within 11–55 days; Fig. 1e). RNA-seq analysis performed on 20 B-ALL cases and 6 age-matched control cases (*ER, Onc, Pax* mice that never developed disease) revealed that 14/34 (41 %) differentially expressed genes were components of canonical B cell development pathways (p = 1.26 × 10^−6^; Ingenuity Pathway Analysis), while GSEA revealed a significant enrichment for genes up-regulated in early B-cell development, specifically the pre-B stage (Additional file 2: Figure S1). Perturbation of B-cell homeostasis, in particular a maturation arrest at the pro-/pre-B stage, is a hallmark of human B-ALL [9]. Thus, our mouse model and the human disease show significant similarities, both in terms of differentially expressed genes and the stage of B-cell arrest. Interestingly we did not find that *Pax5* heterozygosity accelerated leukemia development (Fig. 1a), suggesting its sole contribution to B-ALL development in our model is at the level of the induction of maturation arrest. This is in contrast to an additional cross we performed in which *Etv6*^*+/RUNX1-SB*^ mice were bred to an *Ink4a*-deficient background resulting in a significantly decreased latency to leukemogenesis (p = 0.0012 using a Log-rank test; Additional file 3: Figure S2), which is in agreement with reports that *INK4A* inactivation is associated with an aggressive clinical course in *ETV6-RUNX1*+ B-ALL [10].

**Fig. 2:**
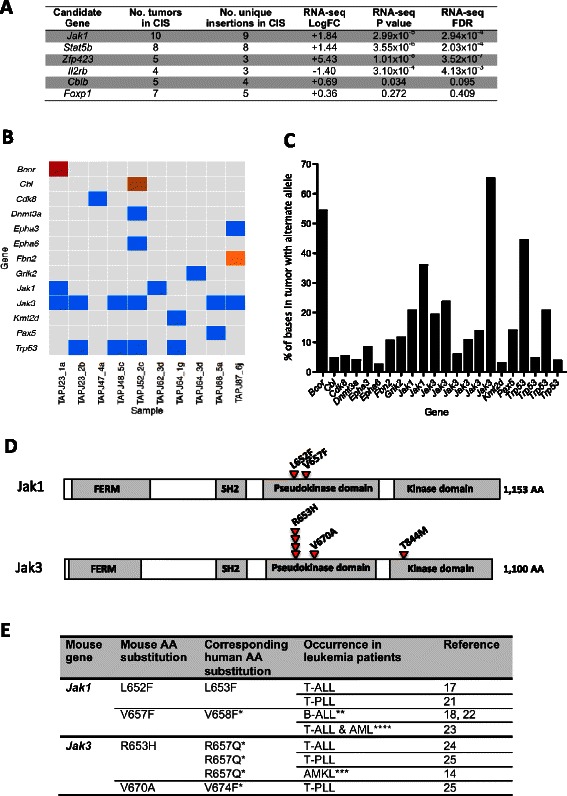
Common insertion site and somatic mutation analysis of BCP-ALL cases in *Etv6-RUNX1, T2Onc, Pax5* mice. **a** Transposon common insertion sites (CIS) in B-ALL cases. All CIS shown have a genome-wide p value of <0.025. Alterations in expression levels were determined using RNA-seq data (from 20 *ER, Onc, Pax* mice that developed B220+, CD19+ BCP-ALL and 8 age-matched ‘control’ *ER, Onc, Pax* mice that never developed disease), whereby the expression level of a gene in BCP-ALL cases containing insertions in a particular CIS was compared to the expression level of that gene in ‘control’ cases. FC, log fold change; FDR, false discovery rate (significance was assessed at an FDR of 5 %). **b** Acquired somatic events in B-ALL cases in *ER, Onc, Pax* mice. Each column represents a sample (individual mouse). *Red* indicates a stop mutation, *brown* indicates a splice acceptor variant, *yellow* indicates a splice region variant (intron) and *blue* indicates a missense variant. **c** The fraction of reads (expressed as a percentage) reporting the somatic variant alleles shown in **b** (in the same order as shown in **b**). **d** Schematic diagram of the protein structure of Jak1 and Jak3, showing the locations of the mutations identified in this study. FERM, band 4.1 ezrin; SH2, src-homology domain. **e** Somatically acquired mutations in Jak1/3 found in *ER, Onc, Pax* B-ALL tumors in which the corresponding JAK1/3 mutations have been found in human cancers. AA, amino acid. ^*^These mutations have been shown to have gain-of-function and/or transforming activity. ^**^These cases were pediatric high-risk B-cell progenitor ALL excluding BCR-ABL1+ ALL and hypodiploid ALL. ^***^Acute megakaryoblastic leukemia of Down syndrome. ^****^An AML with t(15;17)(q22;q12)

To define common transposon insertion sites (CISs), loci in the genome that have increased clustering of transposon insertion events and hence may contain candidate driver genes, tumor DNAs from the 20 mice that developed B220+ CD19+ B-ALL with a tumour cell fraction >60 % were analysed using 454-based ligand-mediated PCR sequencing [7]. Six statistically significant CISs were identified: *Jak1*, *Stat5b*, *Zfp423*, *Il2rb*, *Cblb* and *Foxp1* (Fig. 2a). Four of these 6 genes (*Zfp423*, *Cblb, Stat5b* and *Foxp1*) have well-characterised roles in regulating B-cell maturation. Analysis of the RNA-seq data generated from the tumor collection confirmed that insertions in *Zfp423, Jak1* and *Stat5b* resulted in significantly increased expression of these genes (Fig. 2a). Increased *ZNF423* expression has been reported in BCP-ALL (revealing a strong association with *ETV6-RUNX1*+ cases) and elevated expression of this gene has been linked to a B-cell differentiation block [11]. Activation of the JAK/STAT signaling pathway is a frequent theme in hematological malignancies. In fact, increased expression of activated STAT5 is correlated with poor prognosis in ALL patients, and haploinsufficiency of *Pax5* or *Ebf1* synergize with constitutively expressed *STAT5* to induce B-ALL [12]. Somatic mutations of *CBL/CBLB* in B-ALL typically involve small deletions affecting the intron/exon boundaries of exon 8, leading to skipping of this exon and the abolition of E3 ligase function; the transposon insertions in *Cblb* were in intron 8 and thus are likely to function via a similar mechanism [13]. Mutation of CBL is an alternative route to activate the RAS pathway [13], and mutations in *RAS* have been reported in hyperdiploidy BCP-ALL and *ETV6-RUNX1*+ cases [14]. Thus, our *Etv6-RUNX1*;* Pax5*^*+/−*^ mouse model has several cardinal genetic features associated with B-ALL.

 Interestingly an *Etv6+/RUNX1-SB*, *T2Onc*^*+/+*^*, Pax5*^*+/−*^ mouse (TAPJ23.1a), in which transposition was not occuring, developed transplantable B-ALL (Fig. 1e), suggesting a contribution of background somatic mutations to tumor development leading us to investigate the somatic mutation landscape by targeted exome sequencing of 404 established cancer genes and candidate B-ALL drivers in 17 B220 + CD19+ B-ALL cases (Fig. 2b; Additional file 1). Strikingly the most commonly mutated genes were *Jak3* (6/17 mice, 35 %), *Trp53* (4/17 mice, 23 %) and *Jak1* (2/17 mice, 11 %) with the missense mutations in *Jak1/3* predominantly located in the pseudokinase domain (Fig. 2d). This domain has been demonstrated to exert an important negative regulatory function on the kinase domain [15] with many of the amino acid changes we identified falling into positions of *JAK1/3* reported as being mutated in human leukemias, and shown to confer gain-of-function or transforming activity (Fig. 2e) [16–18]. Somatic mutations in *JAK1* and *JAK3* and have been reported in adult B-ALL [19] and high-risk/poor prognosis pediatric B-ALL [20], respectively. The variant allele frequency of *Jak1/3* mutations was around 25 % suggesting that cells with these mutations represent a major clonal fraction (Fig. 2c). Recurrent somatic mutations in *Jak1/3* have recently been reported in B-ALL tumors from *Pax5*^*+/-*^ mice [PMID: 25855603], suggesting that the synergy with Jak mutations in our model is a result of the knockout allele for *Pax5* rather than the presence of the *Etv6-RUNX1* allele. We also observed somatic *Trp53* mutations in our mouse tumors with copy number and/or sequence alterations of *p53* being an independent risk predictor of inferior outcome/high risk of treatment failure in B-ALL patients [21, 22].

## Conclusions

Collectively, our findings support a model in which multiple small defects in a network of factors that regulate B-cell maturation (such as Pax5, Cblb, Zfp423, Foxp1, and Stat5b) together with activation/inactivation of oncogenes/tumor suppressor genes (such as the JAK/STAT signaling pathway and p53) cooperate with *Etv6-RUNX1; Pax5*^*+/−*^ to result in the development B-ALL. Our transplantable B-ALL tumors represent a novel tool for assessing potential therapeutic intervention strategies in cases of high risk/poor outcome B-ALL.

## Additional files

Additional file 1: **List of targeted exome baits.** The file contains a list of genes that were captured as part of the targeted exome sequencing performed as part of this study. (XLSX 53 kb)
Additional file 2: Figure S1. Pathway analysis showing enrichment for genes involved in early B-cell function in our model. (PPT 166 kb)
Additional file 3: Figure S2. Accelerated tumor development in *Ink4a*^−/−^; *Etv6*^*+/RUNX1-SB*^ mice. (PPT 162 kb)
