# Supplementary figures and images for: Somatic drivers of B-ALL in a model of ETV6-RUNX1; Pax5+/− leukemia
Source: BMC Cancer. 2015 Aug 13;15:585. doi: 10.1186/s12885-015-1586-1 (PMC4542115; doi:10.1186/s12885-015-1586-1)

## Slide 1
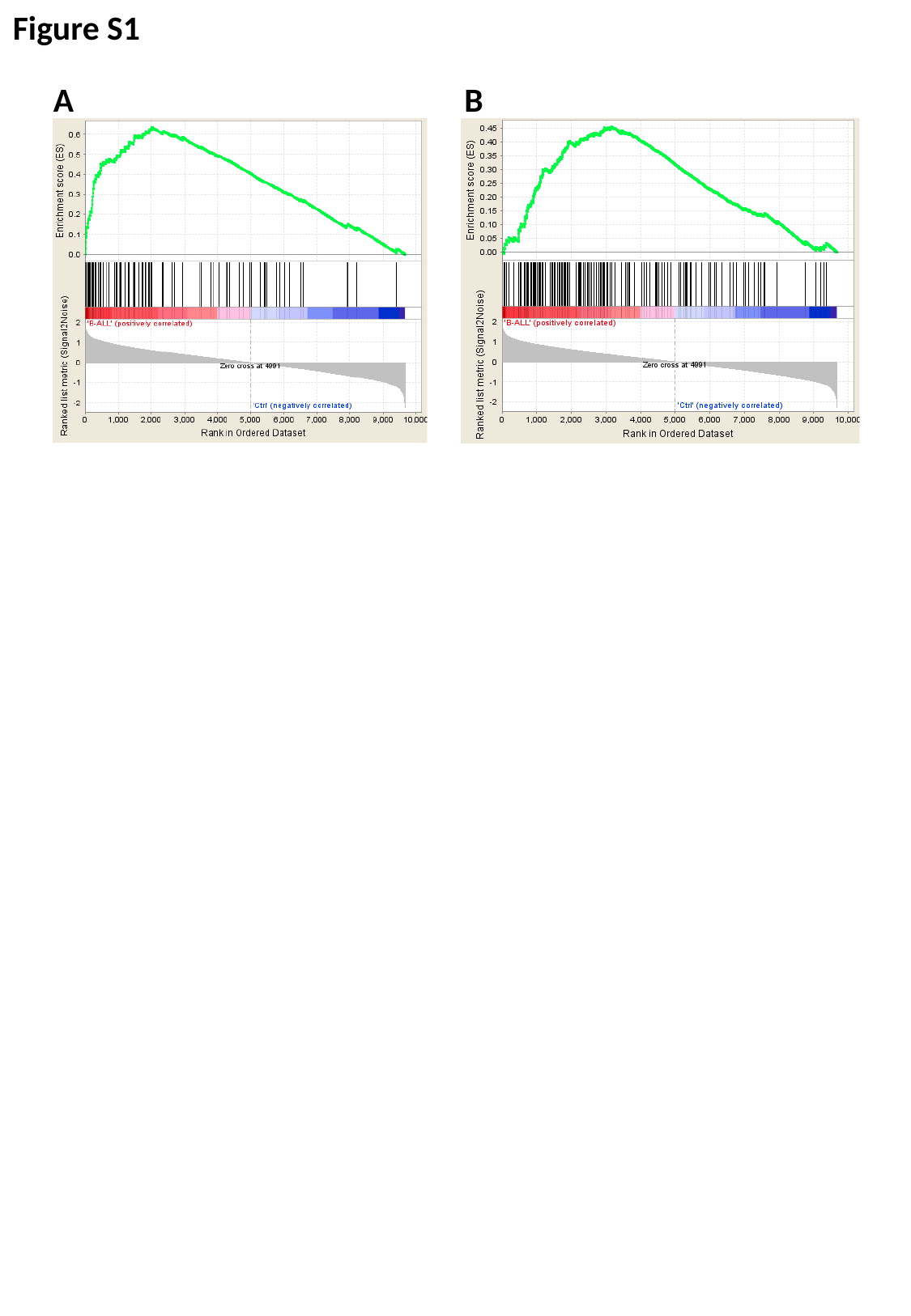

Figure S1
B
A

Supplement: Additional file 2: Figure S1. — Pathway analysis showing enrichment for genes involved in early B-cell function in our model. (PPT 166 kb) [file 12885_2015_1586_MOESM2_ESM.ppt]

## Slide 1
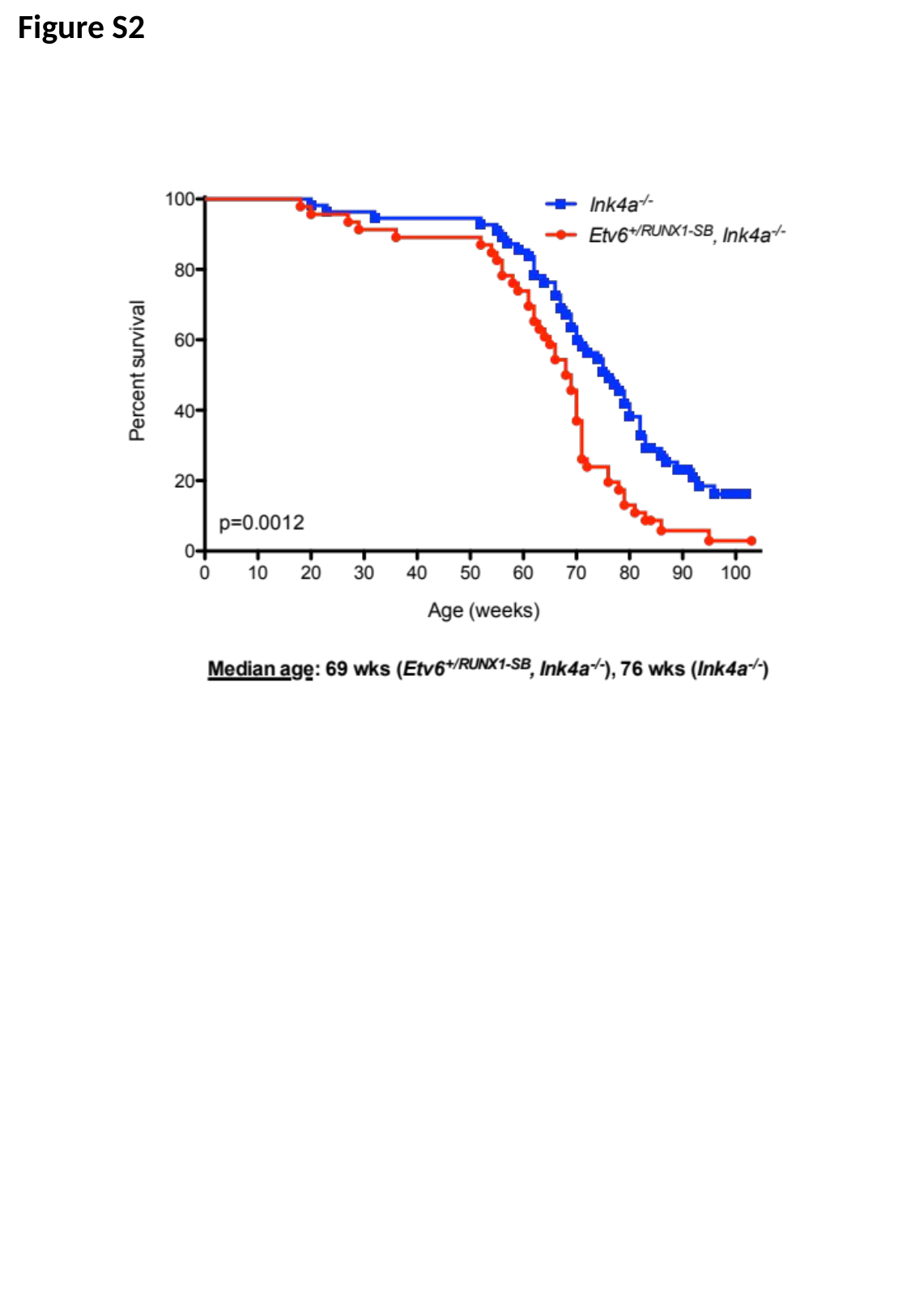

Figure S2

Supplement: Additional file 3: Figure S2. — Accelerated tumor development in Ink4a−/−; Etv6+/RUNX1-SB mice. (PPT 162 kb) [file 12885_2015_1586_MOESM3_ESM.ppt]
